# Histological, hormonal and transcriptomic reveal the changes upon gibberellin-induced parthenocarpy in pear fruit

**DOI:** 10.1038/s41438-017-0012-z

**Published:** 2018-01-03

**Authors:** Lulu Liu, Zhigang Wang, Jianlong Liu, Fengxia Liu, Rui Zhai, Chunqin Zhu, Huibin Wang, Fengwang Ma, Lingfei Xu

**Affiliations:** Institution: College of Horticulture, Northwest A&F University, Taicheng Road NO.3, Yangling, Shaanxi Province China

## Abstract

Phytohormones play crucial roles in fruit set regulation and development. Here, gibberellins (GA_4+7_), but not GA_3_, induced pear parthenocarpy. To systematically investigate the changes upon GA_4+7_ induced pear parthenocarpy, dynamic changes in histology, hormone and transcript levels were observed and identified in unpollinated, pollinated and GA_4+7_-treated ovaries. Mesocarp cells continued developing in both GA_4+7_-treated and pollinated ovaries. In unpollinated ovaries, mesocarp cells stopped developing 14 days after anthesis. During fruit set process, GA_4+7_, but not GA_1+3_, increased after pollination. Abscisic acid (ABA) accumulation was significantly repressed by GA_4+7_ or pollination, but under unpollinated conditions, ABA was produced in large quantities. Moreover, indole-3-acetic acid biosynthesis was not induced by GA_4+7_ or pollination treatments. Details of this GA–auxin–ABA cross-linked gene network were determined by a comparative transcriptome analysis. The indole-3-acetic acid transport-related genes, mainly auxin efflux carrier component genes, were induced in both GA_4+7_-treated and pollinated ovaries. ABA biosynthetic genes of the 9-cis-epoxycarotenoid dioxygenase family were repressed by GA_4+7_ and pollination. Moreover, directly related genes in the downstream parthenocarpy network involved in cell division and expansion (upregulated), and MADS-box family genes (downregulated), were also identified. Thus, a model of GA-induced hormonal balance and its effects on parthenocarpy were established.

## Asian pear: Plant hormones induce seedless fruit

Various hormonal, anatomical and genetic changes explain how a plant hormone can induce pears to make seedless fruit without fertilization. Lingfei Xu and colleagues from Northwest A&F University in Yangling, China, studied the effect of plant growth hormones known as gibberellins on fruit development in the Dangshansuli pear, *Pyrus bretschneideri* Rehd. They found that spraying a mixture of two gibberellins, known as GA_4+7_, on unpollinated flowers produced seedless mature fruits of a normal size, whereas treatment with another gibberellin, GA_3_, yielded fruit that died before harvest. Analyses of gene expression, hormone levels and cell growth in the developing pear fruits showed that applying GA_4+7_ to unpollinated flowers generated many of the same effects as pollination. The findings could aid in future commercial production of this popular Asian fruit.

## Introduction

For most plants, fruit set and development are triggered by pollination and fertilisation, which are highly sensitive to environmental conditions^1^, and unsuitable temperatures, rain and high wind speeds negatively affect pollination^2^. Most fruit species, including pear, apple and cherry, exhibit natural self-incompatibility, and at least two inter-compatible cultivars are necessary to guarantee successful pollination in an orchard, which wastes land and resources. Parthenocarpic fruit set is independent of pollination and, therefore, does not require pollinizers. Previous studies showed that phytohormones, such as auxins and gibberellins (GAs), can promote parthenocarpy in several species^3–7^. Mesejo et al.^8^ showed that GA promotes cell division in ovary walls, causing fruit set. In addition to these hormones, other phytohormones, such as cytokinins, ethylene and abscisic acid (ABA), also take part in fruit set^9^. However, the precise roles of these hormones in regulating parthenocarpic fruit set and development are still poorly understood.

At present, the molecular mechanisms of parthenocarpic fruit set are still unclear in woody fruit trees, with only a few components related to this process having been uncovered. Two auxin response factors (ARFs), namely *ARF7* and *ARF8* had been shown associate with parthenocarpy. Transgenic tomato with decreased *SlARF7* levels formed parthenocarpic fruit^10^, and a natural parthenocarpic eggplant mutant showed that *ARF8* is downregulated in buds compared with in wild-type plants^11^. Thus, both *ARF7* and *ARF8* transcription factors are important negative regulators during fruit set. Auxin/indole-3-acetic acid (Aux/IAA) proteins are transcriptional regulators involved in auxin regulated plant development, and a loss-of-function *IAA9* mutant tomato formed parthenocarpic fruit^12^, suggesting that *IAA9* is also a negative regulator of auxin involved in regulating parthenocarpic fruit set. GA signalling in particular plays an important role in regulating parthenocarpic fruit set because the increased auxin content in ovaries produced by parthenocarpic tomato is mediated through GA^13^. Moreover, tomato mutants *pat* and *pat-2* increase the expression levels of GA biosynthetic genes, increasing the GA content, which forms parthenocarpic fruit^14^. The GA 20-oxidase (*GA20ox*) family of genes catalyse inactive GA to active GA, and overexpressing the citrus gene *CcGA20ox1* in tomato led to the development of parthenocarpic fruits^15^. In addition, *GA2ox*s are a type of catabolic enzyme that deactivate active GA. The silencing of all five *GA2ox* genes in transgenic tomato plants led to a significant increase in the GA_4_ content, and the plants exhibited the ability to develop parthenocarpically^16^. DELLA proteins are important negative regulators in the GA signalling pathway that restrict growth and development. Silencing of *SlDELLA* in tomato can induce parthenocarpic fruits^17^. GA depletes the DELLA protein^18,19^ and releases the repression of GA-response genes.

In addition, silencing of a tomato floral organ-specification gene, *TM29*, a SEPELLATA-like MCM1, AGAMOUS, DEFICIENCE and SRF (MADS) box gene, also caused seedless fruits^20^. The loss-of-function mutation of *MdPI*, a MADS-box transcription factor, produces parthenocarpic fruit in apple^21^. Recently, the MADS box gene *AGL6* was shown to regulate parthenocarpy in tomato, and loss function of SlAGAMOUS-LIKE 6 result in tomato facultative parthenocarpy^22^.

Most cultivated pears are self-incompatible; therefore, understanding the mechanisms of GA-induced parthenocarpic fruit would have great benefits for both production practices and theoretical studies. Here we studied changes upon gibberellins (GA_4+7_)-induced parthenocarpic pear fruit set at the histological, hormonal and transcriptome levels, which have been little studied previously. The aims of the study were: (1) to evaluate the effects of exogenous gibberellic acid (GA_3_) and GA_4+7_ on parthenocarpic fruit set in ‘Dangshansuli’ (*Pyrus bretschneideri* Rehd.) to determine whether GA_4+7_, or GA_3_, could induce parthenocarpy in pear; (2) to observe the ovaries of unpollinated, pollinated and a 75 mg L^−1^ GA_4+7_ treatment of ‘Dangshansuli’ during early stages using morphological and histological methods to explore the similarities and differences among these treatments; (3) to measure gibberellins, auxin and abscisic acid levels to study the hormone balance effects in fruit set; and (4) to perform an RNA sequencing (RNA-Seq) analysis of the ovary transcriptomes at three stages, 3, 9 and 14 days after anthesis (DAA), to study the molecular regulatory mechanism of GA_4+7_-induced parthenocarpy in pear.

## Materials and methods

### Plant materials and experimental treatments

Experiments were carried out in a pear orchard located in Meixian, Shaanxi Province, China (34.29°N, 107.76°E, and 514 m above sea level). The average annual precipitation is 574.6 mm, and the average annual temperature is 12.7 °C.

Eight treatments, the unpollinated (control), 25, 50 and 75 mg L^−1^ GA_3_, 25, 50 and 75 mg L^−1^ GA_4+7_, and hand pollinated, were applied to 15-year-old ‘Dangshansuli’ trees, which were grafted onto *P*. *betulifolia* Bge rootstocks. Three branches of each treatment were used as three replicates. Two days before anthesis, all of the treatments and the control were bagged to prohibit pollination. Water was sprayed on unpollinated flowers as the unpollinated treatment. Solutions of GA_3_ alone at 25, 50 and 75 mg L^−1^, and GA_4+7_ alone at 25, 50 and 75 mg L^−1^, were sprayed on unpollinated flowers of “Dangshansuli” at anthesis. Hand pollination was carried out at the same time. Then, 30 fruits per treatment were randomly sampled for the determination of hormone levels, morphological and cytological observations, and RNA sequencing at each sampling time point. Flowers/fruits were collected at 0, 3, 9, 14 and 153 DAA.

A part of each sample was immediately fixed in formaldehyde-acetic acid-alcohol for histological observation, while the rest of the samples were frozen in liquid nitrogen and then stored at −80 °C for further assays.

### Determination of fruit set rate

A total of 30 blooms on each branch were labelled and bagged immediately after treatments. At 20 DAA, the bags were removed. The formula used to calculate the fruit set rate was as follows:

$${\mathrm{Fruit}}\,\,{\mathrm{set}}\,\,{\mathrm{rate}}( \% ) \\ = ( {{\mathrm{Number}}\,\,{\mathrm{of}}\,\,{\mathrm{fruitlets}}\,\,{\mathrm{remaining}}/30} ) \times 100.$$

### Phytohormone analysis

Samples of 0.2 g were ground in an ice-cold mortar with 8 mL of 80% (v/v) methanol extraction medium that contained 1 mM butylated hydroxytoluene as an antioxidant 22. The extracts were incubated at 4 °C for 4 h and then centrifuged at 3500 r.p.m. for 8 min at 4 °C. The supernatants were passed through Chromosep C18 columns (C18 Sep-Pak Cartridge; Waters Corporation, Millford, MA, USA), which were washed with 10 mL of 100% and 5 mL of 80% (v/v) methanol. The hormone sediments were dried under nitrogen gas and then dissolved in 2 mL of phosphate-buffered saline (PBS) containing 0.1% (v/v) Tween 20 and 0.1% (w/v) gelatine (pH 7.5).

Phytohormones were separately analysed using GA_1+3_, GA_4+7_, IAA and ABA ELISA Kits produced by the Phytohormones Research Institute, China Agricultural University, China. Yang’s method for quantitative phytohormone determination was used^23^.

### Paraffin sectioning

To conduct the histological observation, fruit samples of unpollinated, pollinated and GA_4+7_ treatments were collected at 3, 9 and 14 DAA, immediately fixed in formaldehyde-acetic acid-alcohol fixative^24^ and stored at 4 °C. The ovaries were dehydrated in an ethanol/xylene series and embedded in paraffin, 10 s into 8-µm-thick slices, dried and stained with safranin and fast green^25^. The anatomical images were observed using a microscopic imaging system (BX51 + PD72 + IX71, OLYMPUS, Japan).

### Transcriptome analysis

The total isolated RNAs were used for RNA-Seq on an Illumina HiSeq 2500. The sequencing library is prepared by random fragmentation of the cDNA sample, followed by 5′ and 3′ adapter ligation. Adapter-ligated fragments are then PCR amplified and gel purified. Sequences were screened for primer concatemers, weak signal and poly A/T tails. Low-quality reads were eliminated based on the score value (reads with >30% of bases with quality score (Q value) of <20) and the remaining high quality reads were filtered for short reads below 50 bp. Adaptors were first trimmed, and then reads were further assembled by GS de novo assembler (v2.6)^26^. Singletons cleaning using Seqclean and lucy with a parameter of minimum length 100 bp Illumina hiseq reads produced in paired-end formats (101 bp) were also assembled using the Trinity software package^27^. Reads were filtered and trimmed, and then mapped onto ‘Dangshansuli’ (*P*. *bretschneideri* Rehd.) coding sequences using the SOAP aligner^28^.

Clean reads were mapped using bowtie 1.1.2 to generate read alignments for each sample^29^. The gene expression level was calculated by fragments per kilobase of exon model per million mapped reads^30^. The RNA-Seq data of unpollinated ovaries were used as the controls. A false discovery rate of <0.001 and an absolute value of |log_2_ ratio| > 1 were used as the thresholds for the significance of differentially expressed genes (DEGs). Genes were annotated using the ‘Dangshansuli’ database (http://www.ncbi.nlm.nih.gov/genome/?term=pyrus) as a reference. Three independent biological replications were sequenced and analysed.

### Quantitative real-time PCR validation of gene expression levels

The qRT-PCR was performed on a Life Technologies (ABI) machine using the SYBR Premix Ex Taq Kit (TaKaRa, Dalian, China). The cDNA templates were reverse transcribed using total RNA extracted from ovaries of unpollinated, pollinated and GA_4+7_ treatments at 3, 9 and 14 DAA. Then, 11 selected genes were used to verify the expression results. *Actin* was used as the internal reference for the gene expression analysis. The use actin gene as internal reference is *Actin 7* gene. All of the primers for selected genes were designed using Primer Premier 5.0 software, and are listed in Supplementary Table S2. The PCR reactions were carried out using an initial incubation at 95 °C for 30 s, and then 40 cycles of 95 °C for 5 s and 60 °C for 30 s. All reactions were performed on three biological and three technical replicates. Relative quantification of specific mRNA levels was performed using the cycle threshold (Ct) 2^−ΔΔCt^ method.

### MapMan analysis of DEGs

To study the DEGs involved in carbohydrate and photosynthesis metabolism between pollinated and unpollinated, and GA_4+7_-treated and pollinated, we used the online web software Mercator to obtain the pear protein annotation mapping file for MapMan with the default parameters^31^. MapMan was then used to classify the DEGs into functional categories^32^.

### Statistical methods

The data were statistically analysed using an analysis of variance and tested for significant (*P* *<* 0.05) treatment differences using Duncan’s test.

## Results

### GA_4+7_ induced parthenocarpy in ‘Dangshansuli’

**Fig. 1:**
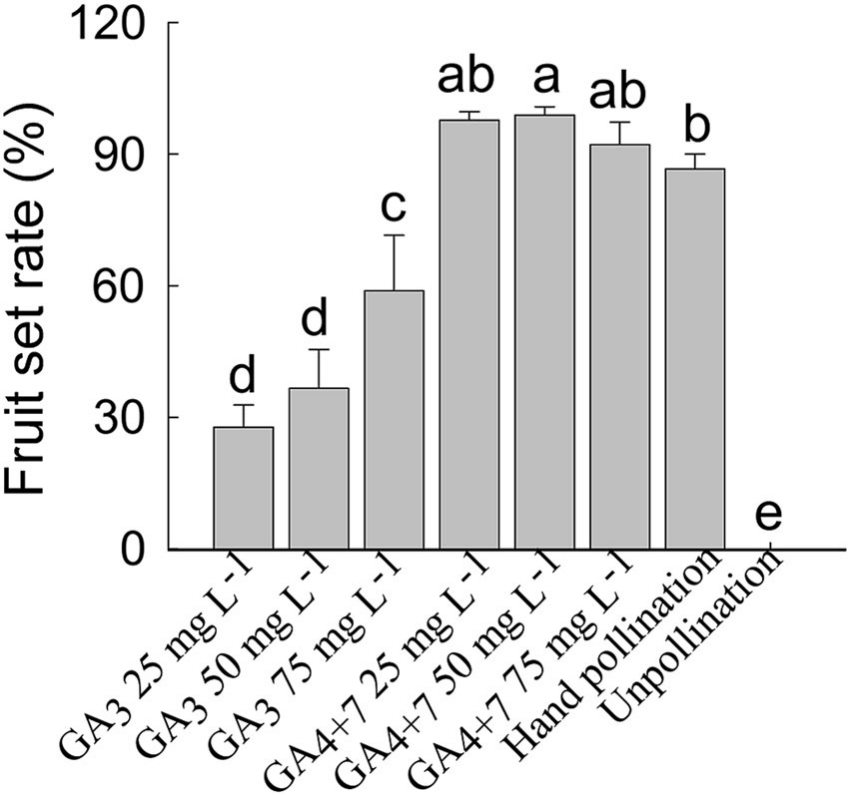
The effects of gibberellins on ‘Dangshansuli’ pear fruit set. Fruit set rate in response to unpollination, pollination and different concentrations (from 25, 50 and 75 mg L^−1^) of GA_3_ and GA_4+7_ (from 25, 50 and 75 mg L^−1^) at 20 DAA. Values of fruit set were means of three replicates with 30 ovaries per replicate (±SD). Significant differences (*P* < 0.05) between treatments are showed by different letters according to Duncan’s test

The fruit set rate reached 86.67% for hand pollination, 88.24–98.85% for different concentrations of GA_4+7_ treatments, and no fruit set for unpollinated flowers. Although the highest set rate among the GA_3_ treatments was 58.89%, which was lower than those of the GA_4+7_ treatments (Fig. 1), all of the GA_3_-treated fruits were eventually lost before harvest, while GA_4+7_ induced seedless mature fruits of a normal size. Thus, GA_4+7_, rather than GA_3_, may play an important role during the pear fruit set process.

### Morphological and histological observations in GA_4+7_-induced parthenocarpic fruits

**Fig. 2:**
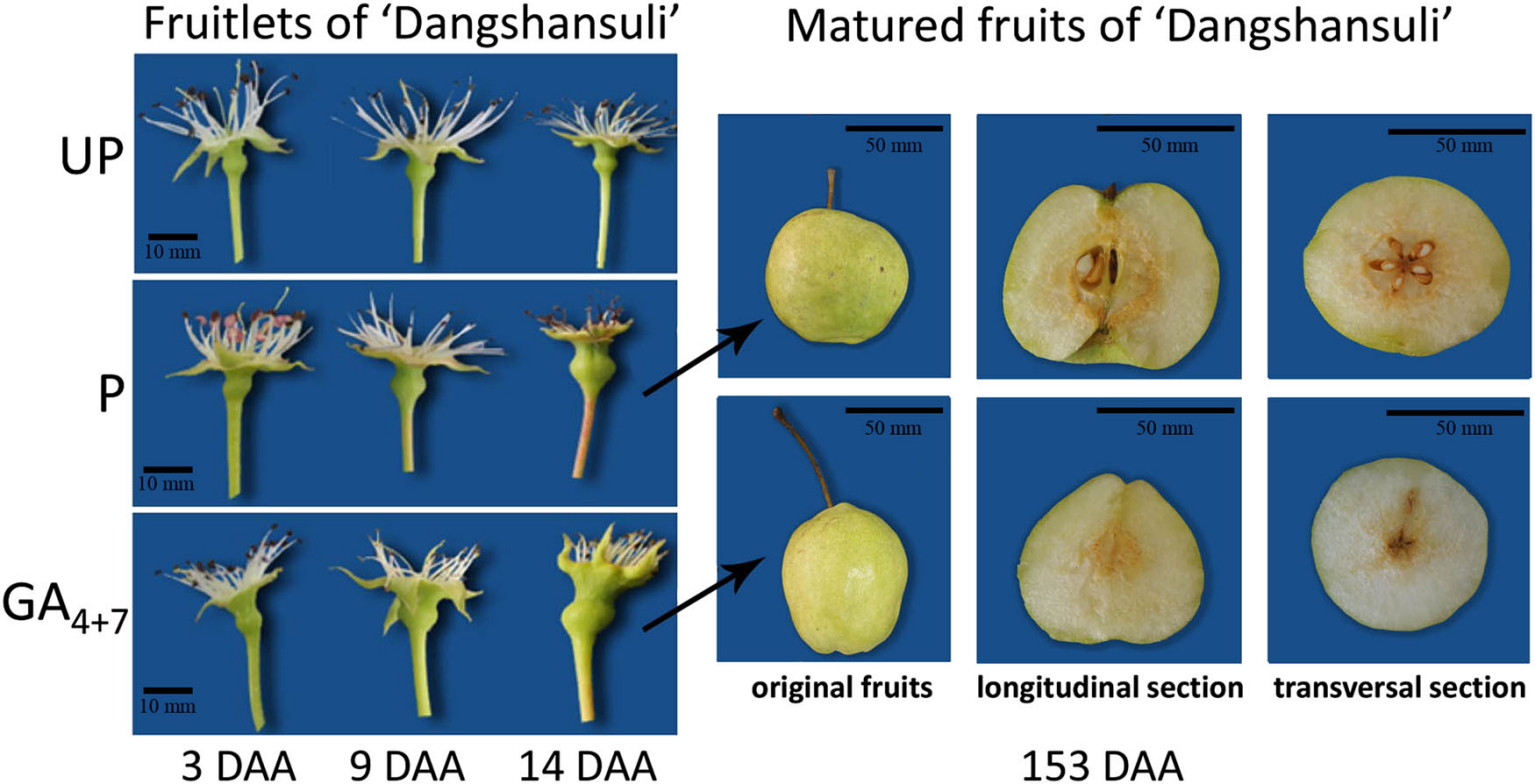
Morphological observation of ‘Dangshansuli’ pear fruits. UP, Unpollination; P, Pollination; GA_4+7_, GA_4+7_ 75 mg L^−1^; fruits were collected at 3, 9, 14 and 153 DAA

Morphological observations showed that unpollination, pollination and GA_4+7_ (From this point of the manuscript, the use of GA_4+7_ will refer to the 75 mg L^−1^ concentration) treated fruits at 3, 9 and 14 DAA, mature fruits developed at 153 DAA. However, the unpollinated ovaries wilted at 14 DAA (Fig. 2). GA_4+7_ induced seedless fruits developed similar size as seeded fruits, but developed smaller core and elongated longitudinal diameter compared to seeded fruits.

**Fig. 3:**
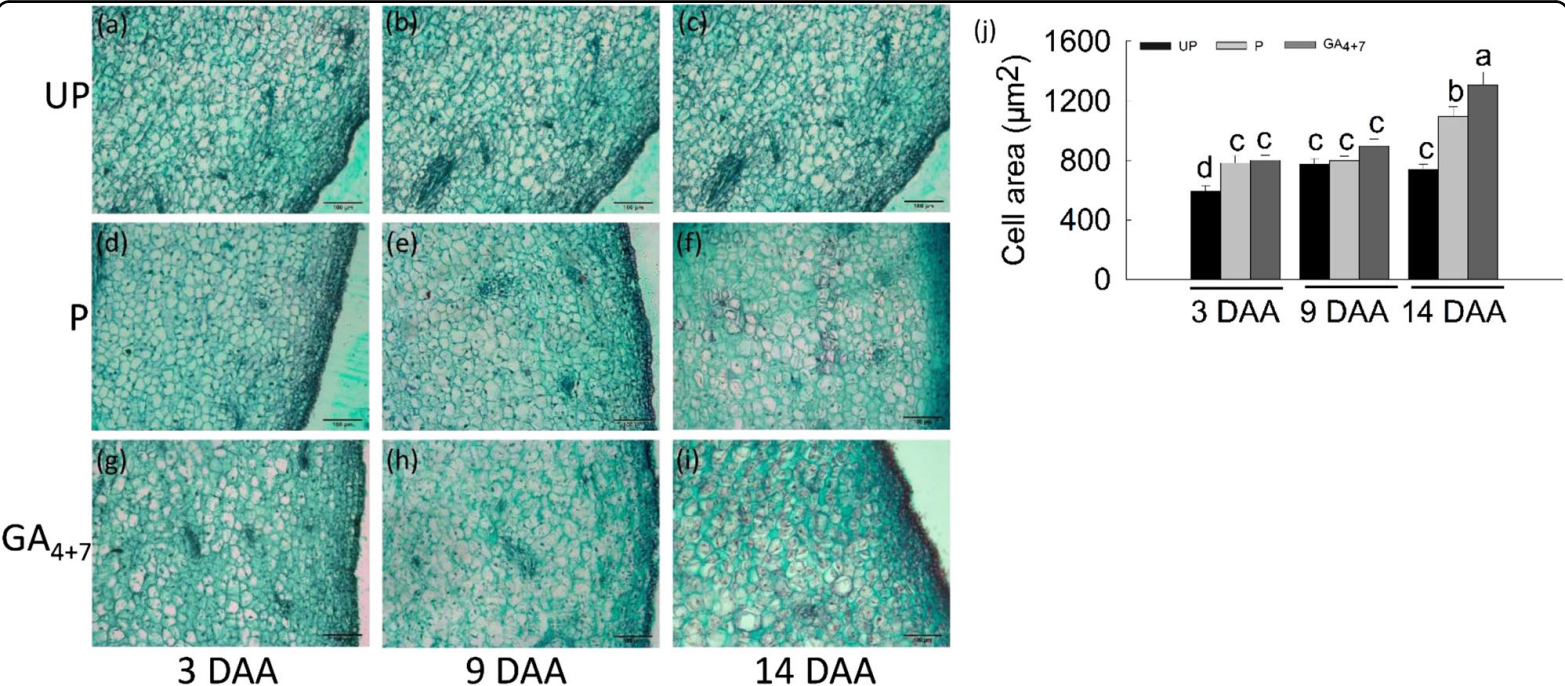
Histological observation of the ‘Dangshansuli’ pear pericarps during the early development stages. **a**, **d**, **g** Microscopic longitudinal sections of the pericarps of unpollination, pollination and GA_4+7_ 75 mg L^−1^ treated fruits at 3 DAA, respectively. **b**, **e**, **h** Microscopic longitudinal sections of the pericarps of unpollination, pollination and GA_4+7_ 75 mg L^−1^ treated fruits at 9 DAA, respectively. **c**, **f**, **i** Microscopic longitudinal sections of the pericarps of unpollination, pollination and GA_4+7_ mg L^−1^ treated fruits at 14 DAA, respectively. **j** Respective quantifications of cell size in mesocarp. UP, Unpollination; P, Pollination; GA_4+7_, GA_4+7_ 75 mg L^−1^. Significant differences (*P* < 0.05) between treatments are showed by different letters according to Duncan’s test. Bar = 100 μm

**Fig. 4:**
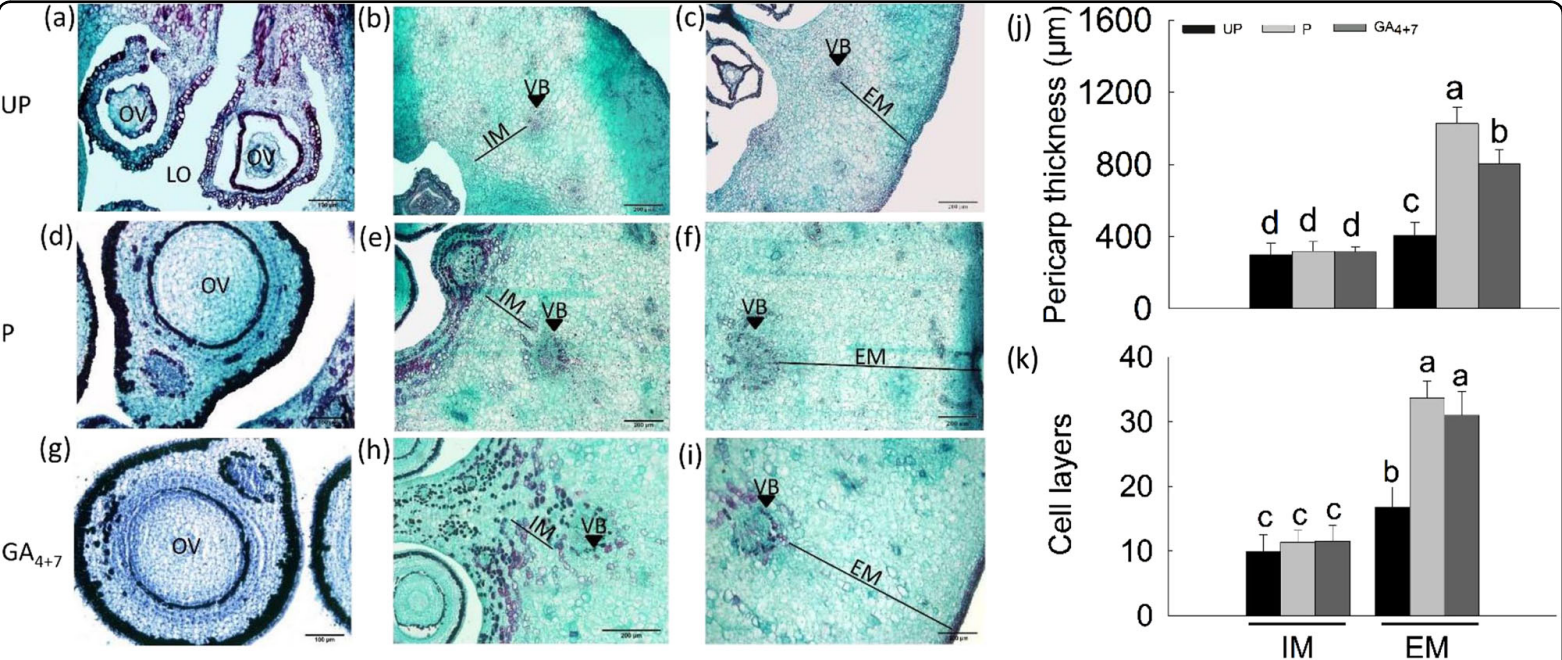
Histological observation of the ‘Dangshansuli’ pear pericarps and ovules. **a**, **d,** **g** Ovule transversal sections at 14 DAA in unpollination, pollination and GA_4+7_ treated fruits. **b**, **c**, **e**, **f**, **h**, **i** Pericarp transversal sections at 14 DAA in unpollination, pollination and GA_4+7_ 75 mg L^−1^ treated fruits, respectively, and the respective of pericarp thickness (**j**) and cell layers (**k**). EM, external mesocarp; IM, internal mesocarp; VB, vascular bundle; LO, locule; OV, ovule. UP, Unpollination; P, Pollination; GA_4+7_, GA_4+7_ 75 mg L^−1^. Significant differences (*P* < 0.05) between treatments are showed by different letters according to Duncan’s test. Bar = 200 μm

Longitudinal sections of mesocarps exhibited GA_4+7_-induced cell expansion from 3 DAA (Fig. 3g) and formed larger cells than pollinated ovaries at 14 DAA (Fig. 3j). Cytological observations showed that apoptosis occurred in the unpollinated ovules and that locular cavities expanded (Fig. 4a), while both pollination and GA_4+7_ induced ovules development (Fig. 4d, g). Cell division process was activated during fruit set as well, transverse sections of mesocarps indicated that pollinated and GA_4+7_-treated ovaries developed thicker external mesocarps (EM, located between the vascular bundles and epidermis) with more cell layers than unpollinated ovaries (Fig. 4). The internal mesocarp (IM, between the vascular bundles and endocarp) did not show any differences among the treatments (Fig. 4). In addition, both pollinated and GA_4+7_-treated fruits developed larger vascular bundles compared with unpollinated ovaries (Fig. 4).

### Hormones involved in GA_4+7_-induced parthenocarpy

**Fig. 5:**
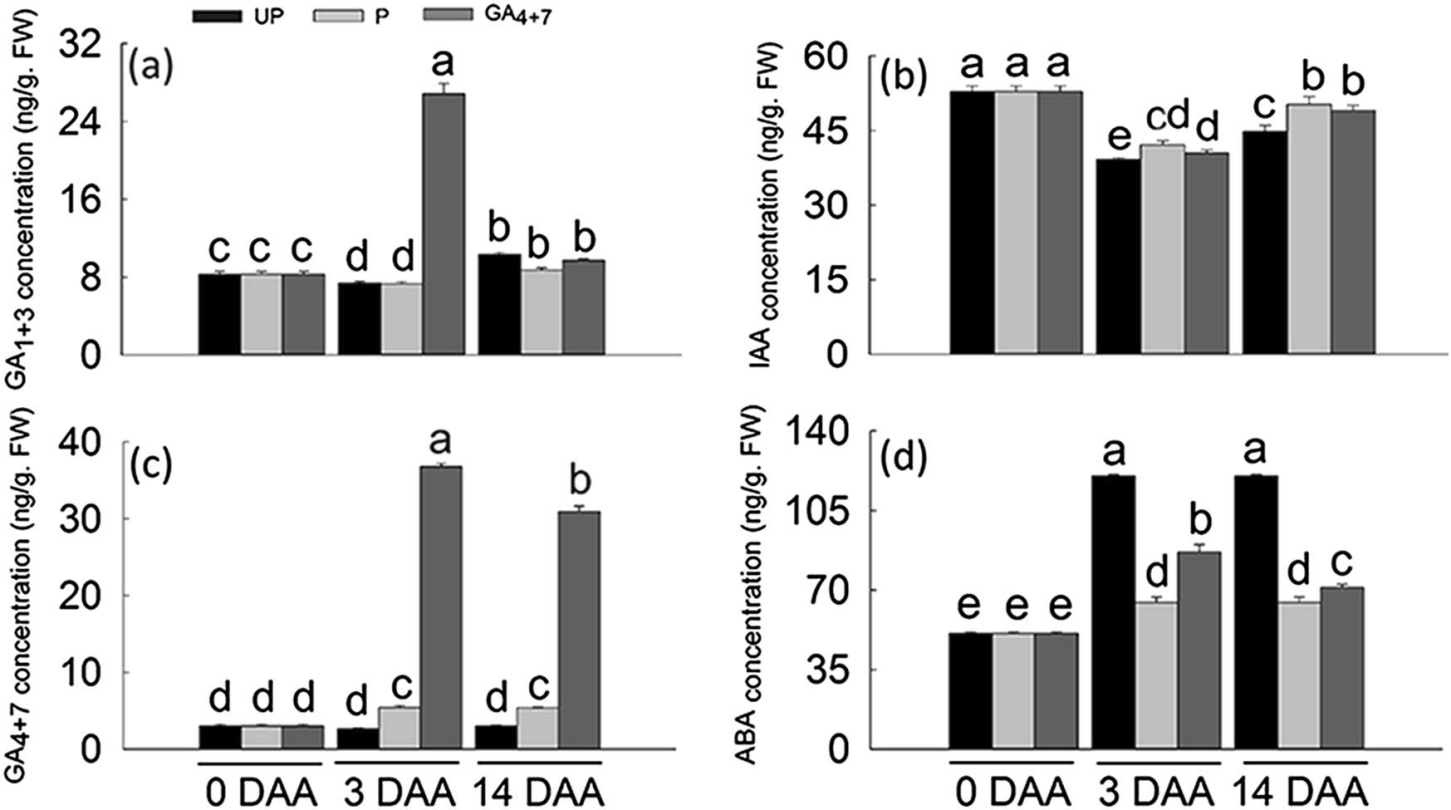
Hormones measurement of ‘Dangshansuli’ pear fruits. The concentrations of plant hormones GA_1+3_ **a**, IAA **b**, GA_4+7_ **c** and ABA **d** in pear ovaries during early development stages (0, 3 and 14 DAA). UP, Unpollination; P, Pollination; GA_4+7_, GA_4+7_ 75 mg L^−1^. Values of hormones were means of three biological and technical replicates (±SD). Significant differences (*P* < 0.05) between treatments are showed by different letters according to Duncan’s test

The GA_4+7_ treatment dramatically and significantly increased GA_1+3_ and GA_4+7_ concentrations at 3 DAA. At 14 DAA, GA_1+3_ content of treated samples dropped to the same levels as unpollinated ovaries, while GA_4+7_ amount was still significantly higher. GA_1+3_ levels showed no significant differences between unpollination and pollination at same developmental stages, but pollination significantly increased the GA_4+7_ concentrations at both 3 and 14 DAA (Fig. 5a, c). IAA levels decreased all significantly from 0 to 14 DAA, while both GA_4+7_ and pollination treatments exhibited higher levels of IAA than unpollination at 3 DAA and 14 DAA (Fig. 5b). ABA levels significantly and dramatically increased in unpollinated flowers at 3 DAA and 14 DAA; however, both pollinated and GA-treated ovaries showed lower levels of ABA at same development points. There is higher level of ABA in GA_4+7_ treatment than pollination at both 3 and 14 DAA (Fig. 5d).

### Transcriptome analysis of pear ovaries during fruit set

Based on our previous experiments, we found that at 14 DAA, the unpollinated flowers began to become withered, so we thought it was a key stage for fruit set. Besides, according to our pre-experiments, we found that hormone levels changed dramatically at 9 DAA, and 3 DAA was the earliest stage for fruit set, so we choose these three stages for further study, in order to more systematically clarify the mechanism of GA_4+7_ induced parthenocarpic fruit set. Ovaries for RNA-Seq were collected from unpollinated, pollinated and GA_4+7_-treated (without pollination) ‘Dangshansuli’ at 3, 9 and 14 DAA. Three independent biological replications were sequenced and analysed.

To investigate changes in the transcriptome during fruit set, we conducted Illumina HiSeq 2500 125PE sequencing of unpollinated, pollinated and GA_4+7_-treated ovaries at 3, 9 and 14 DAA. There were at least 2.86 million raw reads for each sample, except for one sample (GA_4+7__rep3, 65% of the clean reads were mapped to the reference pear), >75% of the clean reads were mapped to the reference pear (*P*. *bretschneideri* Rehd.) genome (Supplementary Table S1). A false discovery rate <0.001, and |log_2_ (ratio)| > 1 were used as the thresholds to determine the significance of DEGs. To verify the RNA-Seq results, qRT-PCR was conducted for candidate genes. We choose 16 genes which was with low, average and high number of reads in GA_4+7_ treated fruits at 14 DAA, and seven important genes involved in gibberellin pathway were also included. The results exhibited in similar expression tendencies as the sequencing results, suggesting that the RNA-Seq data in this study are reliable (Supplementary Fig. S1).

Venn diagrams displayed the distribution of DEGs in pollinated and GA_4+7_-treated ovaries. At 3 DAA, 177 (8.1% of the total) upregulated and 140 (6.4% of the total) downregulated genes were common to pollinated and GA_4+7_-treated ovaries. Following fruit development, the number of common DEGs increased, with 3029 (35.6% of the total) upregulated and 2555 (30% of the total) downregulated genes being common to the two treatments at 14 DAA (Supplementary Fig. S2).

### DEGs involved in GAs homoeostasis and signalling during fruit set

**Fig. 6:**
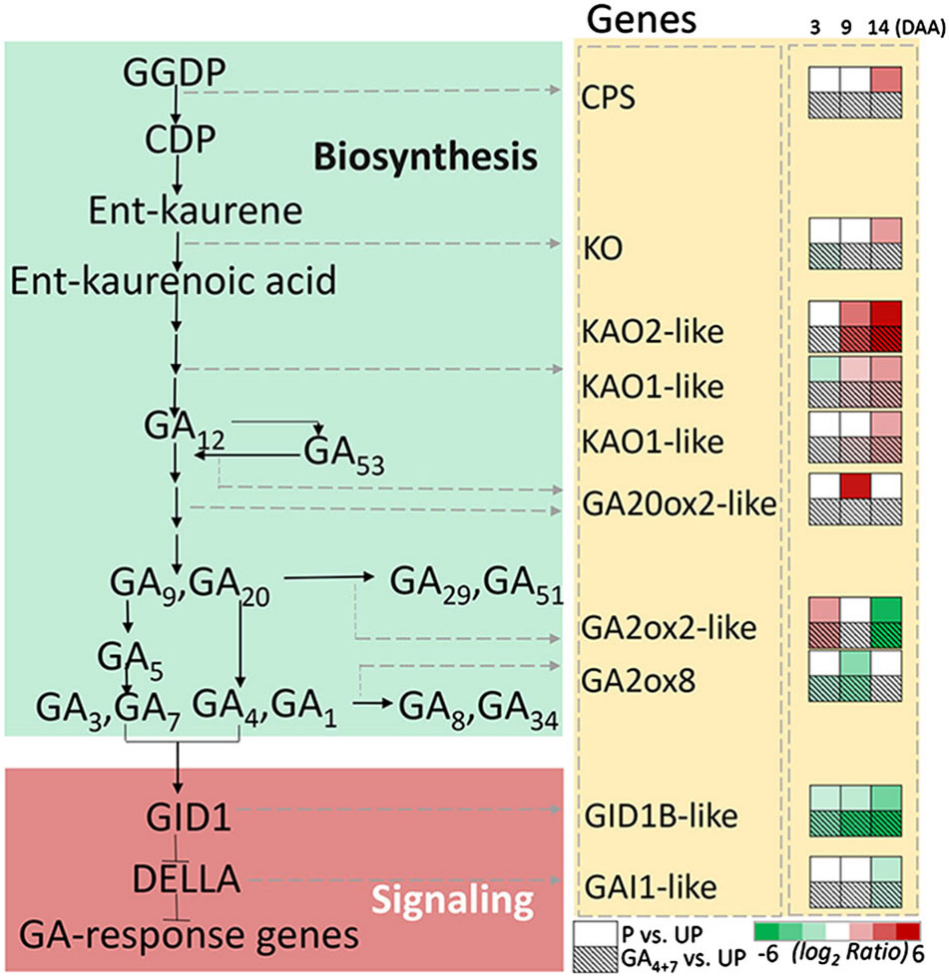
Gibberellin metabolism and signalling pathways following pollination and GA_4+7_ treatments. The number of big rectangle (contain 6 small rectangles) represent copy number of each gene, and the upper small rectangles represent pollination treatment, and the below small squares represent the GA_4+7_ 75 mg L^−1^ treatment, and the small rectangles arrange from left to right represent the expression of genes at 3, 9 and 14 DAA, respectively, with respect to unpollinated ovaries. UP, Unpollination; P, Pollination; GA_4+7_, GA_4+7_ 75 mg L^−1^. Different shades of red and green denote the extent of the change according to the colour bar provided (log_2_ ratio of unpollnation treatment); white indicates no significantly change

GA_4+7_ treatment provided >30 ng/g. FW of exogenous GA_4+7_ (which is more than other treatments), so no more endogenous biosynthesis is needed, thus only ent-kaurenoic acid oxidases (*KAO*s) were induced. In addition to *KAO*s, pollination also induced ent-copalyl diphosphate synthase (*CPS*) (*LOC103957280*) and *GA20ox2-like* (*LOC103960493*) at 14 and 9 DAA, respectively. Genes encoding GA-inactivating *GA2ox*s showed similar expression trends between GA_4+7_ and pollinated treatments. Namely, at 3 DAA, *GA2ox1-like* (*LOC103945984*) was upregulated and then from 9 to 14 DAA exhibited downregulation. In addition, *GA2ox8* (*LOC103931224*) in GA_4+7_-treated ovaries were downregulated at 3 DAA, while the downregulation in pollinated ovaries was delayed to 9 DAA. GA_4+7_ negatively regulated the expression of the GA receptor *GID1* (*LOC103938790*), as well as pollination. The gene encoding DELLA protein *GAI1-like* (*LOC103943039*) was also downregulated in these two treatments at 14 DAA (Fig. 6).

### DEGs involved in auxin and ABA metabolism and their signalling pathways

**Fig. 7:**
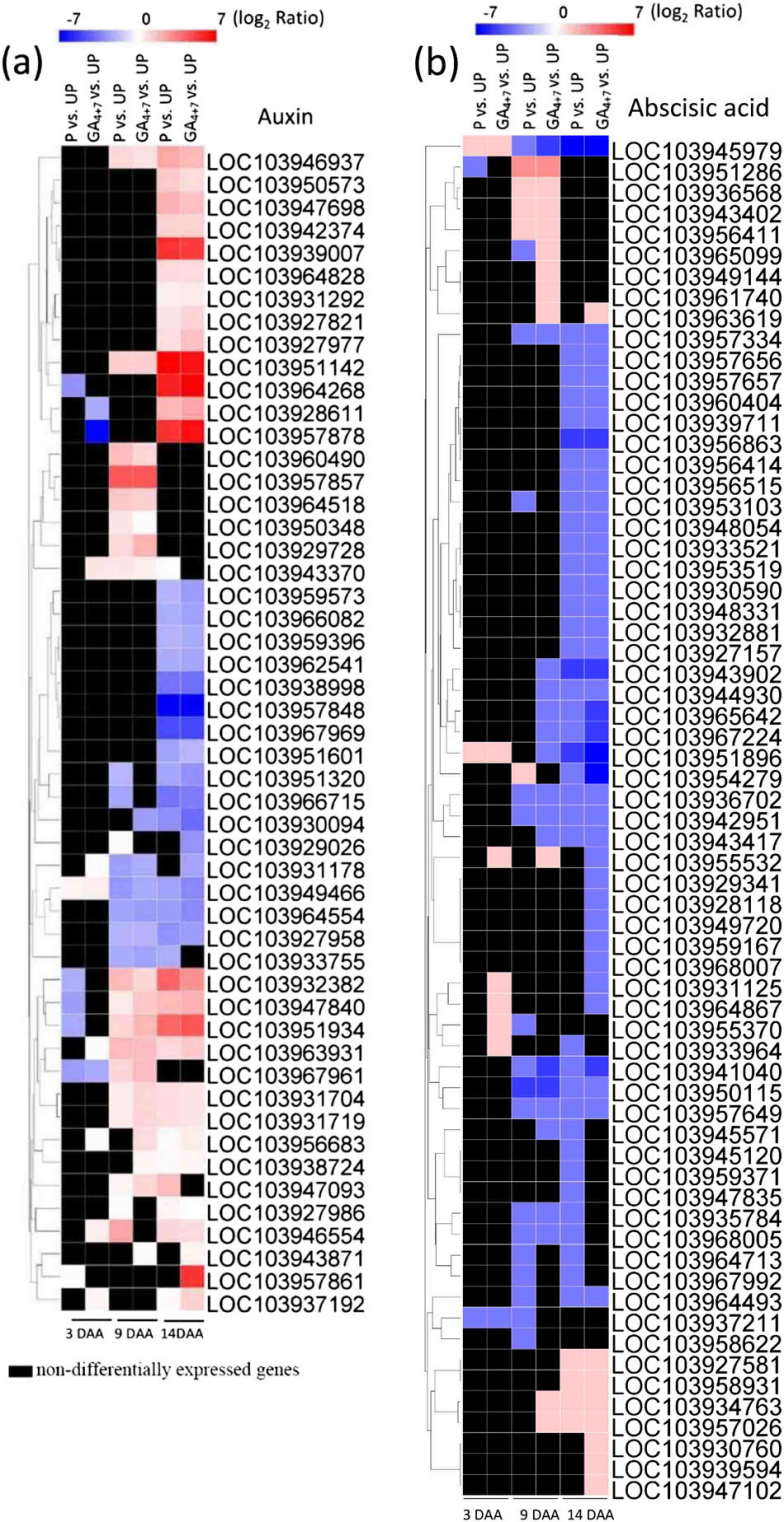
The relative expression of auxin and abscisic acid related genes.1 **a** The relative expression pattern of auxin in pollination and GA_4+7_ 75 mg L^−1^ treated ovaries at 3, 9 and 14 DAA. **b** The relative expression pattern of ABA in pollination and GA_4+7_ 75 mg L^−1^ treated ovaries at 3, 9 and 14 DAA. UP, Unpollination; P, Pollination; GA_4+7_, GA_4+7_ 75 mg L^−1^

Four auxin efflux carrier components were upregulated in GA_4+7_-treated and pollinated ovaries. In particular, the auxin efflux carrier component 6 (*LOC103951142*) was upregulated more than eightfold (log_2_ fold change). DEGs involved in auxin signal transduction were also modified, and *ARF5-like* (*LOC103930094*), *ARF6* (*LOC103951320*), *ARF18-like* (*LOC103962541*) and *ARF19-like* (*LOC103959396*) were downregulated in GA_4+7_-induced parthenocarpic fruits, as well as in the pollinated ovaries (Fig. 7a).

Two genes encoding 9-cis-epoxycarotenoid dioxygenase (*NCED3*) were significantly downregulated in GA_4+7_-treated and pollinated ovaries. In particular, one *NCED3* (*LOC103945979*) was downregulated more than fivefold (log_2_ fold change). A variety of DEGs involved in the ABA signalling pathway were repressed, and 40 protein phosphatase 2Cs were modified during the fruit set process (Fig. 7b). Specific information of genes involved in Fig. 7 was exhibited in Supplementary Tables S6 and S7.

### Cell cycle and expansion-related genes were upregulated during fruit set

**Fig. 8:**
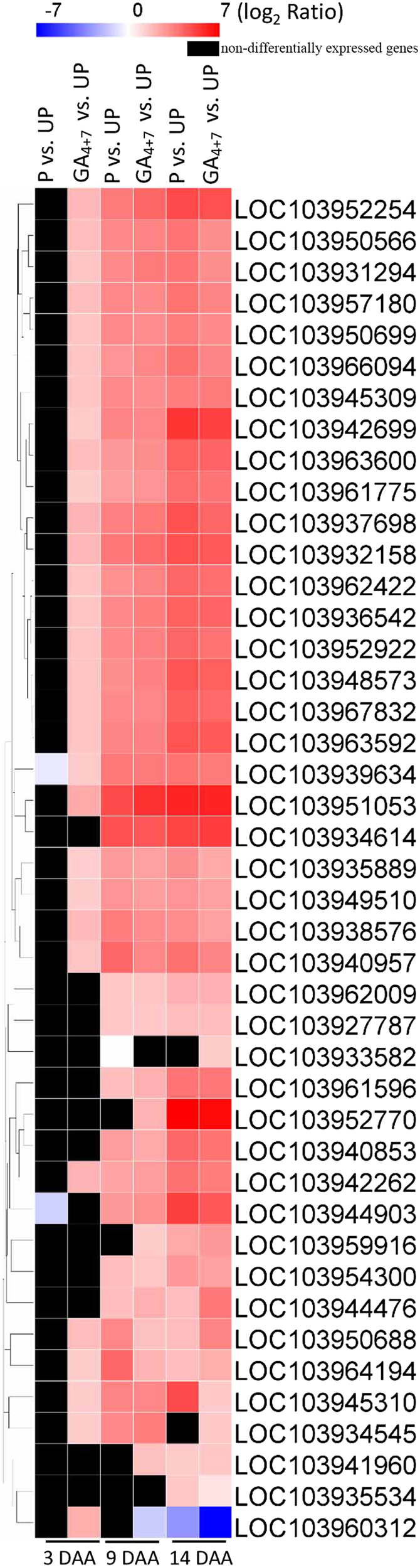
The expression profiles of cyclin and expansin family genes. UP, Unpollination; P, Pollination; GA_4+7_, GA_4+7_ 75 mg L^−1^

Cell division-related and cell wall-related genes were upregulated in GA_4+7_-induced parthenocarpic fruits, as well as in pollinated ovaries. In total, there were 31 upregulated cyclin genes in GA_4+7_-induced ovaries, most of which were significantly upregulated at 3 DAA. However, the upregulation was delayed to 9 DAA in pollinated ovaries. Among these cyclins, a U4-1-like cyclin (*LOC103952770*) was upregulated more than sixfold (log_2_ fold change) in both GA_4+7_-treated and pollinated ovaries at 14 DAA. Nine G2/mitotic-specific cyclins were upregulated in GA_4+7_-induced parthenocarpic fruits from 3 DAA, while their upregulation in pollinated ovaries was delayed to 9 DAA. During fruit development, the elevated levels of these G2/mitotic-specific cyclins increased, and at 14 DAA, all of these G2/mitotic-specific cyclin genes were upregulated more than threefold (log_2_ fold change) in parthenocarpic fruits, as well as in pollinated ovaries. In addition, two cyclin-dependent kinase (CDKs) (*LOC103952922* and *LOC103961775*) genes were upregulated in GA_4+7_-induced parthenocarpic fruits from 3 DAA, while the upregulation in pollinated ovaries was delayed to 9 DAA. RNA-Seq results showed that 10 expansin genes were upregulated, and except for expansin-like B1(*LOC103960312*), in both GA_4+7_-induced parthenocarpic and pollinated ovaries, especially *expansin-A15* (*LOC103951053*), *expansin-B3-like* (*LOC103934614*) and *expansin-A6-like* (*LOC103944903*), which were upregulated more than fourfold (log_2_ fold change) (Fig. 8). In total, 103 cell wall-related DEGs were involved in GA_4+7_-induced parthenocarpic fruits at 3 DAA, and 72 cell wall-related genes were changed by pollination. At later stages, ~140 cell wall-related genes were significantly modified in parthenocarpic and pollinated ovaries (Supplementary Table S3).

### Photosynthetic processes and sugar metabolism were modified during fruit set

**Fig. 9:**
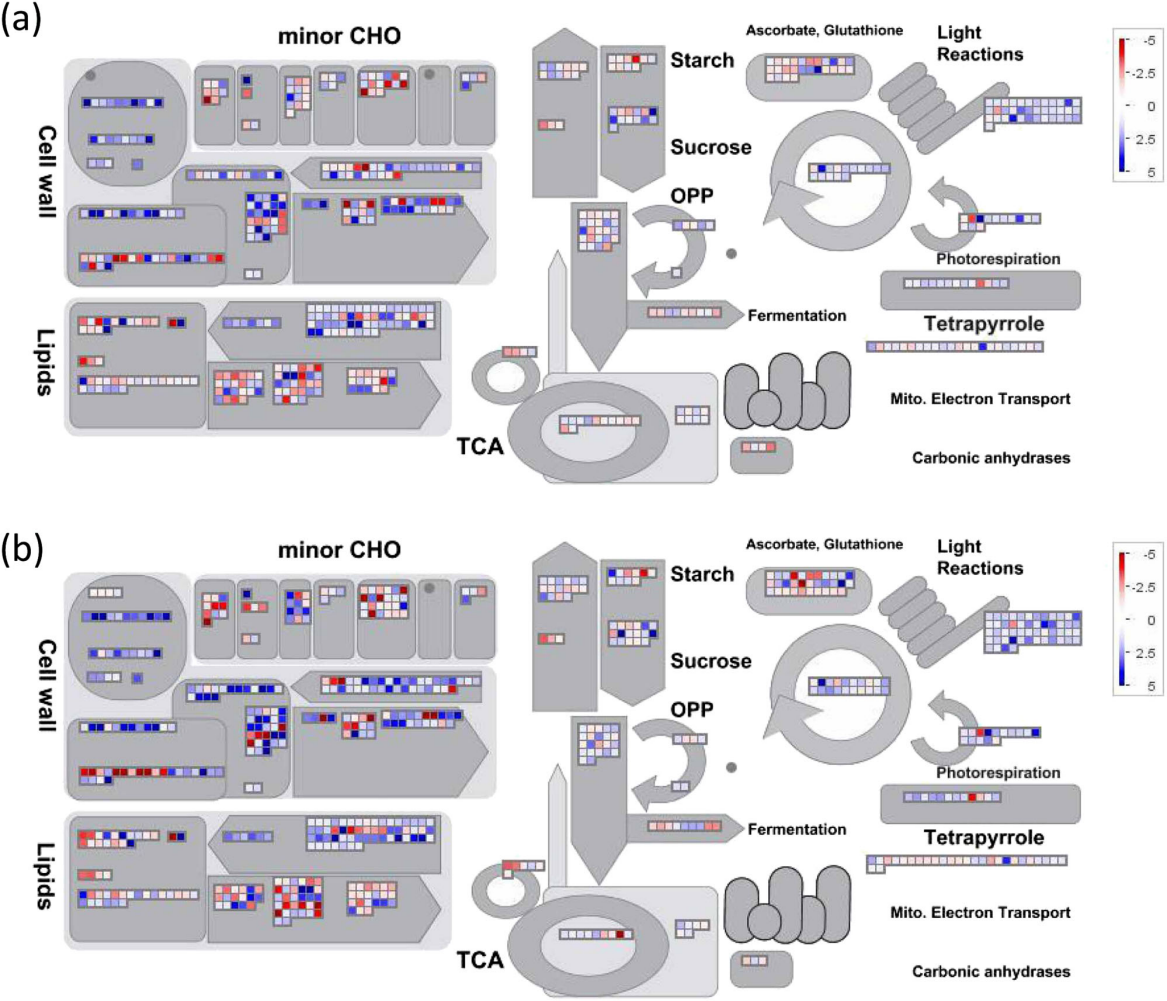
The overview of photosynthesis and carbohydrate metabolism in MapMan pathway. **a** The photosynthesis and carbohydrate metabolism overview’ MapMan pathway was used to visualise the transcriptional changes in pollinated ovaries at 14 DAA. **b** The photosynthesis and carbohydrate metabolism overview’ MapMan pathway was used to visualise the transcriptional changes in GA_4+7_ 75 mg L^−1^ treated ovaries at 14 DAA

To analyse carbohydrate and photosynthesis-related metabolisms, DEGs were grouped based upon their biological functions using MapMan. Results showed that the number of DEGs involved in carbohydrate and photosynthesis metabolisms increased with time going (from 3 to 14 DAA) (Fig. 9, Supplementary Fig. S3). Photosynthesis-related genes were activated, with 59 (Supplementary Fig. S3c) and 45 (Fig. 9a) upregulated DEGs at 9 and 14 DAA, respectively, in pollinated ovaries, and 55 (Supplementary Fig. S3d) and 61 (Fig. 9b) upregulated DEGs in parthenocarpic fruit at 9 and 14 DAA, respectively. At 3 DAA, mainly cell wall-related genes were induced in both pollination and GA_4+7_-treated ovaries. Minor CHO, Lipids, TCA and light reactions processes were activated at 9 DAA, especially for light reactions process, all of DEGs which was involved in this process were upregulated (Supplementary Fig. S3). There were more DEGs involved in these processes at 14 DAA, and most of them were upregulated in both pollination and GA_4+7_-treated fruits.

Four sucrose synthases (*LOC103935320*, *LOC103950289*, *LOC103950290* and *LOC103950291*) were upregulated in parthenocarpic and pollinated ovaries at 14 DAA. Genes encoding elements of the sugar degradation process also changed after GA_4+7_-treatment and pollination. Three sucrose hydrolases (*LOC103959831*, *LOC103961960* and *LOC103963401*) and two alpha amylases (*LOC103950868* and *LOC103955697*) were induced in both parthenocarpic and pollinated ovaries. Two invertases (*LOC103961960* and *LOC103963401*) were upregulated in GA_4+7_-induced parthenocarpic fruits at 9 and 14 DAA, pollination showed similar expression patterns. In particular, the expression of *LOC103961960* was upregulated about eightfold in both parthenocarpic and pollinated ovaries at 14 DAA. Genes involved in cell wall synthesis, such as UDP-glucose 4, 6-dehydratase (*LOC103966239* and *LOC103947469*), UDP-glucose 6-dehydrogenase (*LOC103930880*, *LOC103947238* and *LOC103931361*) and UDP-glucuronic acid decarboxylases (*LOC103944949*, *LOC103948978*, *LOC103928376*, *LOC103928424* and *LOC103930040*) were significantly upregulated in parthenocarpic and pollinated ovaries at 14 DAA, while the expression of three other genes (*LOC103948402*, *LOC103963541* and *LOC103965769*) belonging to the UDP-glucuronic acid decarboxylase family were only found in GA_4+7_-induced parthenocarpic fruits at 14 DAA (Supplementary Table S4).

### Transcription factors (TFs) involved in fruit set

TFs play crucial roles in regulating many developmental processes. There were 124, 240 and 449 DEGs at 3, 9 and 14 DAA, respectively, encoding TFs in GA_4+7_-induced parthenocarpic ovaries, and 59, 199 and 385 DEGs encoding TFs in pollinated ovaries at 3, 9 and 14 DAA, respectively (Supplementary Fig. S4). There were 32, 139 and 329 differentially expressed TFs showed similar expression patterns between GA_4+7_ and pollination treatments at 3 DAA, 9 and 14 DAA, respectively, which shows that the number of TFs increased with time going. Besides, 91, 165 and 177 differentially expressed TFs exhibited differential expression pattern between GA_4+7_ and pollination treated ovaries at 3 DAA, 9 and 14 DAA, respectively. The majority of changed TFs belong to the bHLH, MYB, NAC and WRKY families (Supplementary Fig. S4).

MADS-box genes may play important regulatory roles during fruit development^33^. *AGL19* is one of the MADS-box family genes, both GA_4+7_ and pollination treatments repressed the expression of *AGL19* (*LOC103952886*) at 9 DAA. Besides, *AGL*9 (*LOC103961438*) was only downregulated in GA_4+7_-treated ovaries at 14 DAA. In our analysis, two *DEF*s (*LOC103938026* and *LOC103966740*) were significantly repressed in parthenocarpic ovaries at 14 DAA, while only one *DEF* (*LOC103938026*) was significantly downregulated in pollinated ovaries.

## Discussion

Phytohormones play a crucial role in regulating fruit set, and exogenous applications of plant hormones can induce artificial parthenocarpy^3^. GA_3_ can induce parthenocarpic fruits in several plants, including tomato and grape^34,35^. In our study, exogenous application of GA_4+7_, rather than GA_3_, induced parthenocarpic pear fruit, although exogenous applications of GA_4+7_ could increase the GA_1+3_ content. Pollination increased GA_4+7_ levels, but barely affected the GA_1+3_ content. Thus, GA_4+7_, rather than GA_3_, may play the main role in regulating ‘Dangshansuli’ fruit set. Pollination promoted the expression of *CPS* (except for at 9 DAA), *KO*, *KAO*s and *GA20*oxs, which synthesise bioactive GA, while GA_4+7_ only promoted the expression of *KAO*s. *GA2ox*s, which function to deactivate bioactive GA, most of which were downregulated in parthenocarpic ovaries, as well as in pollinated ovaries. Both GA_4+7_ and pollination repressed the expression of *GID1B-like* from 9 DAA. At 14 DAA, the expression levels of *GAI1-like* were also repressed, and pollination resulted in a similar expression pattern. Thus, GA_4+7_ barely changed the mRNA levels of the encoded biosynthetic GA genes, since GA content was already high to support fruit set processes, while mainly influencing GA-response genes.

Auxins also play key roles during fruit set, and the application of IAA was shown to induce cell division in the tomato pericarp^36^. GA_4+7_ did not increase the IAA concentration, but it promoted auxin efflux carrier components (1c, 1b and 6) expression levels, which was consistent with pollination.

ABA inhibit cell division by inducing *ICK1*, a *CDK* inhibitor^37^, and ABA levels were relatively high in unpollinated ovaries and decreased after pollination. An important ABA biosynthesis gene in tomato, *LeNCED1* was repressed after pollination^38^. Both GA_4+7_ and pollination treatments decreased ABA accumulation in ovaries compared with unpollination, and *NCED3* and *NCED5* were significantly downregulated in these two treatments.

Thus, GA coordinates with IAA and the ABA balance, promoting cell division and expansion, thus stimulating fruit set and development.

Pollination and the following double-fertilisation occurrences in the carpel cause a coordinated sequence of cell division, expansion and differentiation events that result in fruit set and maturity^39^. Exogenous applications of GA_4+7_ promoted the development of unpollinated ovaries, while the unpollinated ovaries ceased to grow and ovules aborted at 14 DAA. Serrani et al.^34^ also found that unfertilised ovules degenerated in the former case in tomatoes. GA_4+7_-treatments produced larger cells than pollination in the mesocarp, which was consistent with GA inducing larger cells than pollination in tomato^34^. However, GA_4+7_ promoted external mesocarp division because GA_4+7_-induced parthenocarpic ovaries had more cell layers than those of the unpollinated ovaries, but similar cell layers as the pollinated ovaries.

Transcriptome data showed that the amounts of cell cycle and cell wall-related genes were modified by GA_4+7_ treatments, suggesting the importance of cell division and cell expansion in fruit set and development. The cell cycle is mainly regulated by two key classes of regulators, *CDK*s and cyclins [8]. A-, B- and D-type cyclins mainly regulate the S and G2/M phases^8,40,41^, and B-type *CDK*s are specifically expressed in G2 and M phase^42^. Our study showed that 33 DEGs, including B-type *CDKs*, A-, B-, D- and U-cyclins and G2/mitotic-specific cyclins, were positively regulated by GA_4+7_ or pollination during fruit set, suggesting that activated G2/M phase regulators are available for cell proliferation. A previous study showed that 14 cell cycle genes were positively associate with cell proliferation during apple fruit set and development^43^.

The cell wall is comprised of cellulose, hemicellulose and pectin, as well as proteins, which influence cell expansion^44^. *Expansin A* (*EXPA*) and *B* (*EXPB*) belong to the α- and β-expansin families, respectively, and many of their members have the ability to induce rapid cell expansion^45^. In our study, *EXPB3-like* and five *EXPA*s were significantly upregulated in GA_4+7_-induced parthenocarpic fruits. Glucanases, other wall hydrolases, and *XET*s affected cell enlargement by regulating cell expansion activities^46^. In our analysis, two *endo-1*,*3*;*1*,*4-beta-D-glucanase-like* genes had decreased expression levels, while four *glucomannan 4-beta-mannosyltransferase* genes had significantly increased expression levels in parthenocarpic and pollinated ovaries. Xyloglucan endotransglucosylase/hydrolase (*XTH*) enzymes play crucial roles in promoting cell expansion by disassembling xyloglucan^47^, and the expression of several *XTH*s was increased, with *XTH*8, *XTH*9, *XTH*31 and *XTH*32 being significantly upregulated in GA_4+7_-induced parthenocarpic ovaries and pollinated ovaries. A variety of cell cycle and cell wall-related genes were significantly upregulated in our analysis, suggesting that these categories of genes also take part in fruit set. UDP-glucuronic acid, a nucleotide sugar that is a precursor of the cell wall, is formed through the activity of UDP-glucose dehydrogenase, which catalyses UDP-glucose into UDP-glucuronic acid^48^. Our analysis showed that both UDP-glucose dehydrogenase and UDP-glucuronic acid decarboxylases were significantly increased in parthenocarpic and pollinated ovaries. This result was consistent with pericarps undergoing rapid cell division and expansion periods, and the activation of cell wall synthesis.

Photosynthesis provides necessary nutrition for fruit and seed set, and deficient photosynthesis is a primary driver of flower, fruit and seed abortion^1^. Antisense silencing of *SlIAA9* induced parthenocarpic tomatoes, which had upregulated expression levels photosynthesis-associated genes^49^, and our analysis corroborated these results. According to the MapMan analysis, the expression levels of photosynthesis-associated genes were upregulated in GA_4+7_-induced parthenocarpic pear ovaries, and pollinated ovaries showed similar results. Plant growth relies on photosynthesis products, mainly in the form of sucrose in most crop species^1^, and invertase cleaves the sucrose to hexose, which promotes fruit and seed set^1^. Our analysis showed that the DEGs involved in sucrose degradation were upregulated in parthenocarpic ovaries, as well as pollinated ovaries. The genes encode enzymes associated with the decomposition of sucrose, suggesting that a high level of energy is demanded during fruit set.

The present study clarified that MADs-box family genes play roles in regulating fruit set^21,50^. Class B MADs-box genes control petal and stamen development, and the loss-of-function of a class B MADs-box results in parthenocarpy in apple, and *DEFICIENS* (*DEF*) belongs to class B MADs-box family^21^. *SlDEF* functions as a repressor in ovules and inhibits the development of ovaries in unpollinated tomatoes, and it was upregulated before or during anthesis in the wild type, but not in the *pat* mutant^51^. In our analysis, *DEF* (*LOC103966740*) was significantly repressed in parthenocarpic and pollinated ovaries. The class E MADS-box genes are essential for all floral whorls involved in specifying organ identities, and *AGL9* is an E class gene^52,53^. Our studies exhibited that *AGL9* was repressed in parthenocarpic fruits.

## Conclusions

**Fig. 10:**
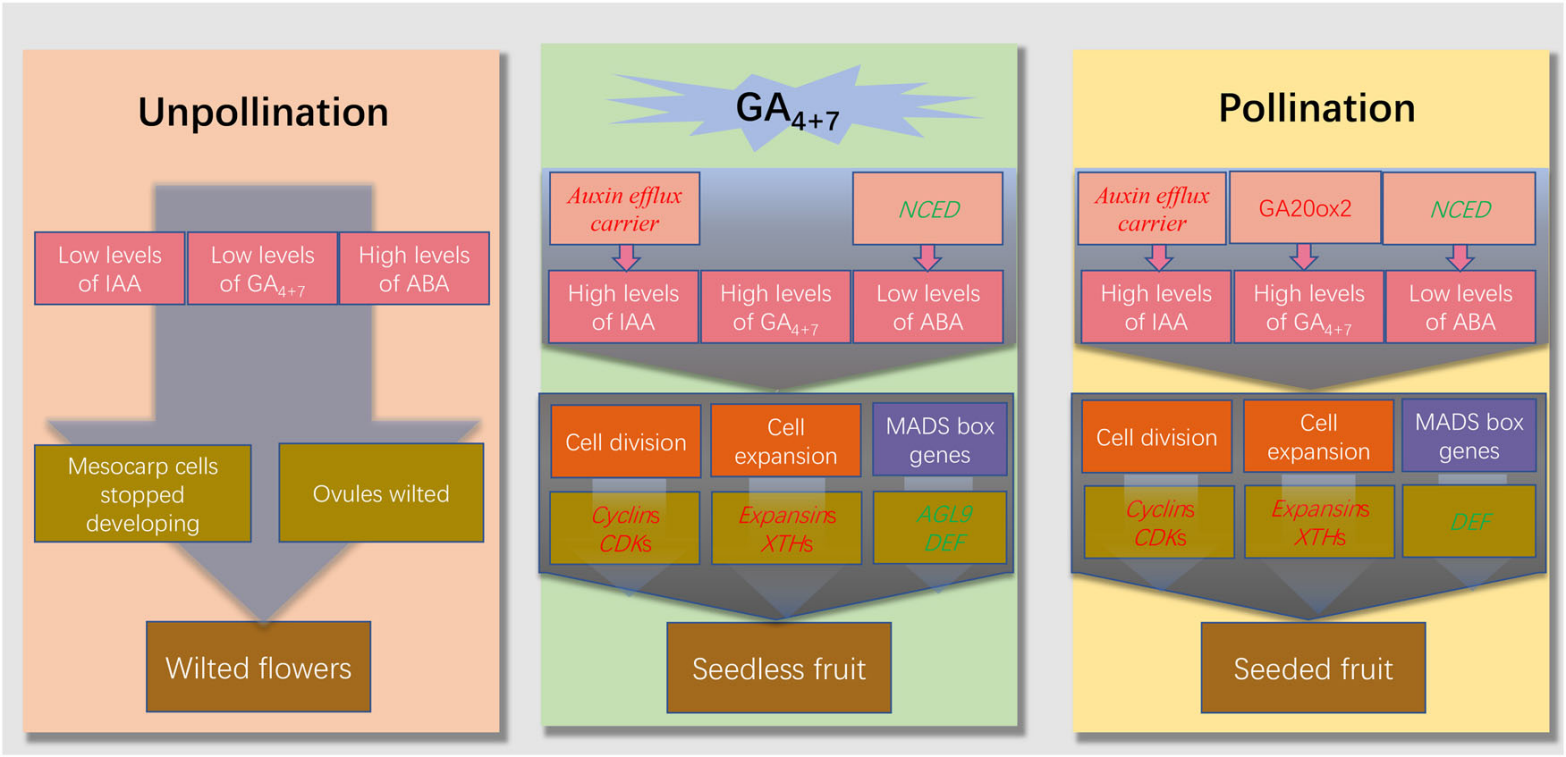
The mechanistic model of fruit set. Gene names in red font represent related genes were upregulated, instead, those in green front represent related genes were downregulated

In summary, our studies found that GA_4+7_, but not GA_3_, induced pear parthenocarpy, which was consistent with pollination increasing the GA_4+7_ contents but not GA_1+3_. GA_4+7_ maintained normal cell growth in mesocarps, as well as pollination, while unpollinated ovaries ceased growing and then shrunk. Genes involved in cell cycle and cell expansion were upregulated in GA_4+7_-induced parthenocarpic pear fruit. RNA-Seq results also revealed that the photosynthesis and sugar metabolism processes were activated, suggesting that they may also take part in fruit set. GA_4+7_ did not increase the IAA level but promoted its transport through gene expression. GA_4+7_ repressed the ABA biosynthetic gene *NCED*, resulting in lower ABA levels in parthenocarpic ovaries. GA_4+7_-repressed genes (MADS-box family genes *AGL9* and *DEF*) were directly related to parthenocarpy. The model presented in Fig. 10 represented that application of GA_4+7_ to unpollinated ovaries may promote the hormonal balance, photosynthesis, sugar metabolism, the cell cycle and cell expansion, and also repress *AGL9* and *DEF* expressions, resulting in fruit set.

## Electronic supplementary material

Supplementary Information

Supplementary Data

## References

[1] Ruan YL, Patrick JW, Bouzayen M, Osorio S, Fernie AR (2012). Molecular regulation of seed and fruit set. Trends Plant Sci..

[2] Ramírez F, Davenport TL (2013). Apple pollination: a review. Sci. Hortic..

[3] Gillaspy G, Ben-David H, Gruissem W (1993). Fruits: a developmental perspective. Plant Cell.

[4] Ding JG (2013). Cytokinin-induced parthenocarpic fruit development in tomato is partly dependent on enhanced gibberellin and auxin biosynthesis. PLoS ONE.

[5] Mesejo C, Reig C, Martínez-Fuentes A, Agustí M (2010). Parthenocarpic fruit production in loquat *Eriobotrya japonica* Lindl by using gibberellic acid. Sci. Hortic..

[6] Niu QF (2015). Effects of exogenous application of GA_4+7_ and N-2-chloro-4-pyridyl-N′-phenylurea on induced parthenocarpy and fruit quality in *Pyrus pyrifolia* ‘Cuiguan’. Plant Growth Regul..

[7] Sugiyama K, Kami D, Muro T (2014). Induction of parthenocarpic fruit set in watermelon by pollination with bottle gourd *Lagenaria siceraria* Molina Standl pollen. Sci. Hortic..

[8] Mesejo C, Yuste R, Reig C, Martínez-Fuente A (2016). Gibberellin reactivates and maintains ovary-wall cell division causing fruit set in parthenocarpic Citrus species. Plant Sci..

[9] Nitsch JP (1952). Plant hormones in the development of fruits. Q. Rev. Biol..

[10] de Jong M, Wolters-Arts M, Feron R, Mariani C, Vriezen WH (2009). The Solanum lycopersicum auxin response factor 7 *SlARF7* regulates auxin signaling during tomato fruit set and development. Plant J..

[11] Du L (2016). *SmARF8*, a transcription factor involved in parthenocarpy in eggplant. Mol. Genet. Genom..

[12] Wang H (2005). The tomato Aux/IAA transcription factor *IAA9* is involved in fruit development and leaf morphogenesis. Plant Cell.

[13] Serrani JC, Carrera E, Ruiz-Rivero O, Gallego-Giraldo L (2010). Inhibition of auxin transport from the ovary or from the apical shoot induces parthenocarpic fruit-set in tomato mediated by gibberellins. Plant Physiol..

[14] Fos M, Proaño K, Nuezc F, García-Martínez JL (2001). Role of gibberellins in parthenocarpic fruit development induced by the genetic system *pat-3/pat-4* in tomato. Plant Physiol..

[15] Greco M, Chiappetta A, Bruno L, Bitonti MB (2012). *Posidonia oceanica* cadmium induces changes in DNA methylation and chromatin patterning. J. Exp. Bot..

[16] Martinez-Bello L, Moritz T, Lopez-Diaz I (2015). Silencing C19-GA 2-oxidases induces parthenocarpic development and inhibits lateral branching in tomato plants. J. Exp. Bot..

[17] Marti C (2007). Silencing of DELLA induces facultative parthenocarpy in tomato fruits. Plant J..

[18] Harberd NP, Belfield E, Yasumura Y (2009). The angiosperm gibberellin-GID1-DELLA growth regulatory mechanism: how an “Inhibitor of an Inhibitor” enables flexible response to fluctuating environments. Plant Cell.

[19] Hirano K (2010). Characterization of the molecular mechanism underlying gibberellin perception complex formation in rice. Plant Cell.

[20] Ampomah-Dwamena C (2002). Down-Regulation of *TM29*, a Tomato SEPALLATA homolog, causes parthenocarpic fruit development and floral reversion. Plant Physiol..

[21] Yao JL, Dong YH, Morris BAM (2001). Parthenocarpic apple fruit production conferred by transposon insertion mutations in a MADS-box transcription factor. Proc. Natl Acad. Sci. USA.

[22] Klap C (2017). Tomato facultative parthenocarpy results from SlAGAMOUS-LIKE 6 loss of function. Plant Biotechnol. J..

[23] Yang JC, Zhang JH, Wang JQ, Zhu QS, Liu LJ (2001). Water deficit-induced senescence and its relationship to the remobilization of pre-stored carbon in wheat during grain filling. Agro J..

[24] Henwood A (2010). Formalin pigment from formaldehyde-acetic acid-alcohol fixed tissues?. J. Histotechnol..

[25] in ZQ (2007). *AtCDC5* regulates the G2 to M transition of the cell cycle and is critical for the function of *Arabidopsis* shoot apical meristem. Cell Res..

[26] Matilla MA, Salmond GPC (2012). Complete genome sequence of serratia plymuthica bacteriophage Ф MAM1. J. Virol..

[27] Haas BJ (2013). De novo transcript sequence reconstruction from RNA-Seq: reference generation and analysis with Trinity. Nat. Protoc..

[28] Li R (2009). SOAP2: an improved ultrafast tool for short read alignment. Bioinformatics.

[29] Langmead B, Trapnell C, Pop M, Salzberg SL (2009). Ultrafast and memory-efficient alignment of short DNA sequences to the human genome. Genome Biol..

[30] Wapinski OL (2013). Hierarchical mechanisms for direct reprogramming of fibroblasts to neurons. Cell.

[31] Lohse M (2014). Mercator: a fast and simple web server for genome scale functional annotation of plant sequence data. Plant Cell Environ..

[32] Thimm O (2004). Mapman: a user-driven tool to display genomics data sets onto diagrams of metabolic pathways and other biological processes. Plant J..

[33] Masiero S, Colombo L, Grini PE, Schnittger A, Kater MM (2011). The emerging importance of type I MADS box transcription factors for plant reproduction. Plant Cell.

[34] Serrani JC, Fos M, Atarés A, García-Martínez JL (2007). Effect of gibberellin and auxin on parthenocarpic fruit growth induction in the cv micro-tom of tomato. J. Plant Growth Regul..

[35] Jung CJ (2014). Gibberellin application at pre-bloom in grapevines down-regulates the expressions of *VvIAA9* and *VvARF7*, negative regulators of fruit set initiation, during parthenocarpic fruit development. PLoS ONE.

[36] Liu X (2016). The role of gibberellins and auxin on the tomato cell layers in pericarp via the expression of ARFs regulated by miRNAs in fruit set. Acta Physiol. Plant.

[37] Wang H (1998). *ICK1*, a cyclin-dependent protein kinase inhibitor from Arabidopsis thaliana interacts with both *Cdc2a* and *CycD3*, and its expression is induced by abscisic acid and its expression is induced by abscisic acid. Plant J..

[38] Nitsch LM (2009). Abscisic acid levels in tomato ovaries are regulated by *LeNCED1* and *SlCYP707A*. Planta.

[39] Vivian-Smith A, Koltunow AM (1999). Genetic analysis of growth regulator-induced parthenocarpy in *Arabidopsis*. Plant Physiol..

[40] Beemster GTS (2005). Genome-wide analysis of gene expression profiles associated with cell cycle transitions in growing organs of *Arabidopsis*. Plant. Physiol..

[41] InzéD VLD (2006). Cell cycle regulation in plant development. Annu. Rev. Genet..

[42] Breyne P (2002). Transcriptome analysis during cell division in plants. PNAS.

[43] Malladi A, Johnson LK (2011). Expression profiling of cell cycle genes reveals key facilitators of cell production during carpel development, fruit set, and fruit growth in apple *Malusxdomestica* Borkh. J. Exp. Bot..

[44] Bashline L, Lei L, Li S, Gu Y (2014). Cell wall, cytoskeleton, and cell expansion in higher plants. Mol. Plant.

[45] Kende H, Bradford K, Brummell D, Cho H, Cosgrove D (2004). Nomenclature for members of the expansin superfamily of genes and proteins. Plant Mol. Biol..

[46] Cosgrove DJ (1997). Relaxation in a high-stress envirsnment: the molecular bases of extensible cell walls and cell enlargement. Plant Cell.

[47] Atkinson RG, Johnston SL, Yauk Y, Sharma NN, Schröder R (2009). Analysis of xyloglucan endotransglucosylase/hydrolase XTH gene families in kiwifruit and apple. Postharvest Biol. Tec..

[48] Klinghammer M, Tenhaken R (2007). Genome-wide analysis of the UDP-glucose dehydrogenase gene family in Arabidopsis, a key enzyme for matrix polysaccharides in cell walls. J. Exp. Bot..

[49] Wang H (2009). Regulatory features underlying pollination-dependent and -independent tomato fruit set revealed by transcript and primary metabolite profiling. Plant Cell.

[50] Ruiu F, Picarella ME, Imanishi S, Mazzucato A (2015). A transcriptomic approach to identify regulatory genes involved in fruit set of wild-type and parthenocarpic tomato genotypes. Plant Mol. Biol..

[51] Mazzucato A, Olimpieri I, Siligato F, Picarella ME, Soressi GP (2008). Characterization of genes controlling stamen identity and development in a parthenocarpic tomato mutant indicates a role for the DEFICIENS ortholog in the control of fruit set. Physiol. Plant.

[52] Theißen G (2001). Development of floral organ identity: stories from the MADS house. Curr. Opin. Biol..

[53] Li XM (2014). Functional conservation and divergence of four ginger *AP1*/*AGL9* MADS-box genes revealed by analysis of their expression and protein-protein interaction, and ectopic expression of *AhFUL* gene in *Arabidopsis*. PLoS ONE.

