# Supplementary material for: Histological, hormonal and transcriptomic reveal the changes upon gibberellin-induced parthenocarpy in pear fruit
Source: Hortic Res. 2018 Jan 3;5:1. doi: 10.1038/s41438-017-0012-z (PMC5798812; doi:10.1038/s41438-017-0012-z)
Supplement: Supplementary file 1 — Supplementary Information [file 41438_2017_12_MOESM1_ESM.pdf]

Supplementary Information

Histological, hormonal and transcriptomic changes reveal the mechanisms of gibberellin-induced parthenocarpy in pear fruit

Lulu Liu, Zhigang Wang, Jianlong Liu, Fengxia Liu, Rui Zhai, Chunqin Zhu, Huibin Wang, Fengwang Ma, Lingfei Xu*

College of Horticulture, Northwest A&F University, Yangling, Shaanxi Province, China

Correspondence:

Lingfei Xu

Email：lingfxu2013@sina.com

Supplementary Figure S1. qRT-PCR verify the RNA-seq results. Relative expression profile of 16 selected genes showed that the expression value in the pairwise comparison among three stages in unpollinated, pollinated and GA_{4+7} mg L^{-1} treated ovaries. Histograms represent expression fold changes as assessed by qPT- PCR, data are reported as means ± SE of three biological replicates (left axis). Lines represent expression fold changes as assessed by RNA-Seq (by using FPKM, right axis). Significant differences (P<0.05) between treatments are showed by different letters according to Duncan's test. UP, Unpollination; P, Pollination; GA_{4+7}, GA_{4+7} 75 mg L^{-1}.

Supplementary Figure S2. Venn diagrams of differently expressed genes (DEGs). The numbers and proportions of DEGs within each group at 3 (a), 9 (b) and 14 DAA (c) are exhibited. UP, Unpollination; P, Pollination; GA_{4+7}, GA_{4+7} 75 mg L^{-1}.

Supplementary Figure S3. The overview of photosynthesis and carbohydrate metabolism in MapMan pathway. (a), the 'photosynthesis and carbohydrate metabolism overview' MapMan pathway was used to visualize the transcriptional changes in pollinated ovaries at 3 DAA. (b), the 'photosynthesis and carbohydrate metabolism' MapMan pathway was used to visualize the transcriptional changes in GA_{4+7} treated ovaries at 3 DAA. (c), the 'photosynthesis and carbohydrate metabolism' MapMan pathway was used to visualize the transcriptional changes in pollinated ovaries at 9 DAA. (d), the 'photosynthesis and carbohydrate metabolism' MapMan pathway was used to visualize the transcriptional changes in GA_{4+7} treated ovaries at 9 DAA.

Supplementary Figure S4. The up/down-regulated transcription factors involved in pollination and GA_{4+7} treated ovaries at 3, 9 and 14 DAA. UP, Unpollination; P, Pollination; GA_{4+7}, GA_{4+7} 75 mg L^{-1}.

Supplementary Table S1. List of mapping results.

Supplementary Table S2. List of qRT-PCR primers.

Supplementary Table S3. Cell wall related genes.

Supplementary Table S4. Photosynthesis and carbohydrate metabolism related genes.

Supplementary Table S5. List of MADS-box family genes.

Supplementary Table S6. Auxin metabolism and signaling pathway related genes.

Supplementary Table S7. Abscisic acid metabolism and signaling pathway related genes.

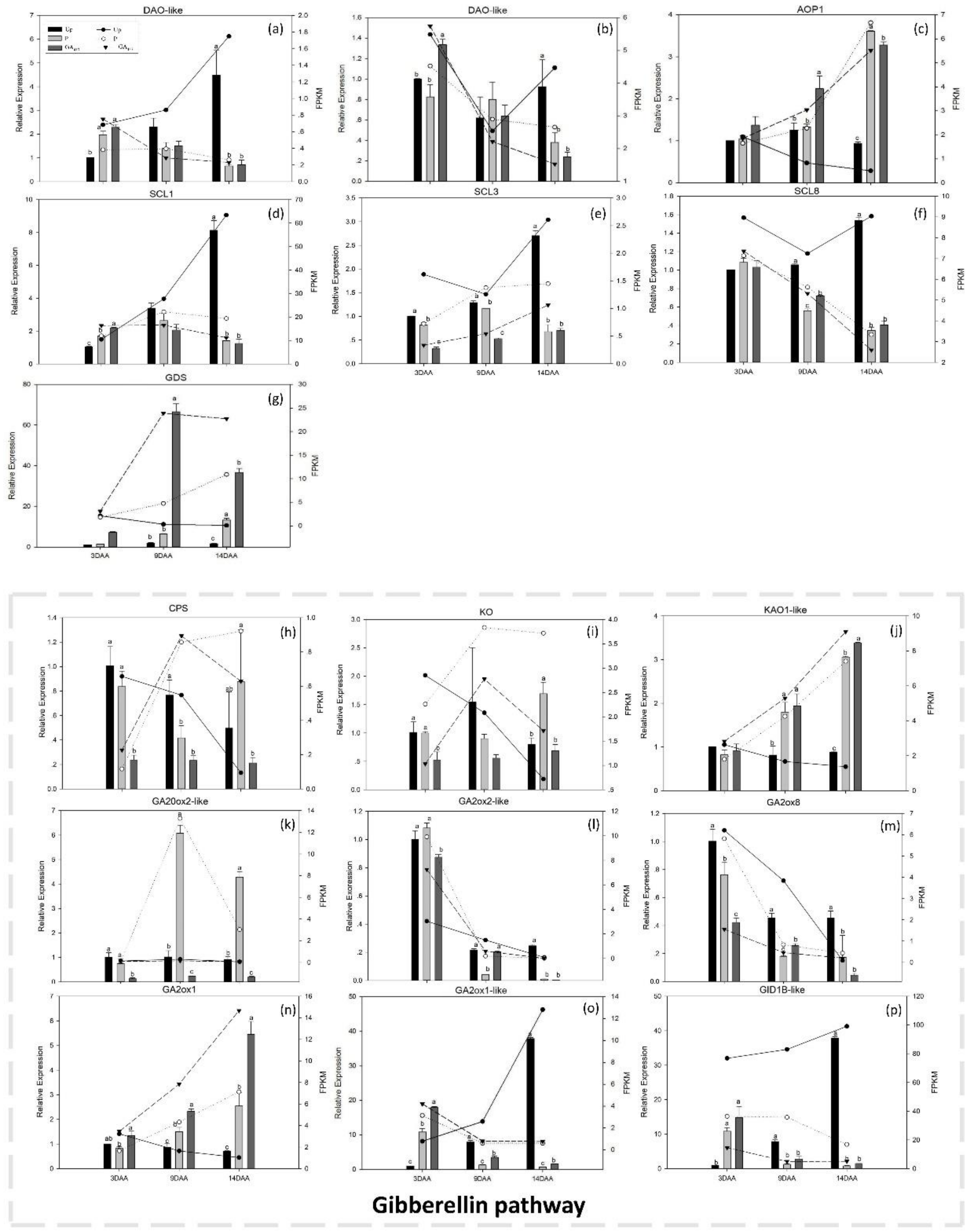

Supplementary Figure S1. qRT-PCR verify the RNA-seq results. Relative expression profile of 16 selected genes showed that the expression fold change in the pairwise comparison among three stages in unpollinated, pollinated and GA_{4+7} mg L^{-1} treated ovaries. Histograms represent expression fold changes as assessed by qPT- PCR, data are reported as means ± SE of three biological replicates (left axis). Lines represent expression fold changes as assessed by RNA-Seq (by using FPKM, right axis). Significant differences (P<0.05) between treatments are showed by different letters according to Duncan's test. UP, Unpollination; P, Pollination; GA_{4+7}, GA_{4+7} 75 mg L^{-1}.

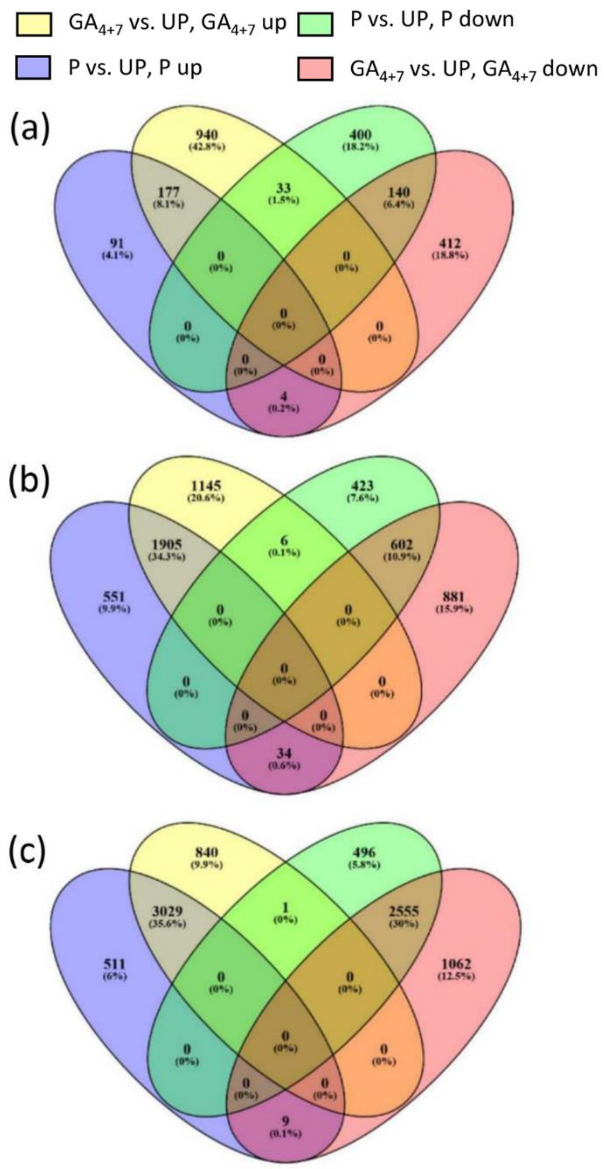

Supplementary Figure S2. Venn diagrams of differently expressed genes (DEGs). The numbers and proportions of DEGs within each group at 3 (a), 9 (b) and 14 DAA (c) are exhibited. UP, Unpollination; P, Pollination; GA_{4+7}, GA_{4+7} 75 mg L^{-1}.

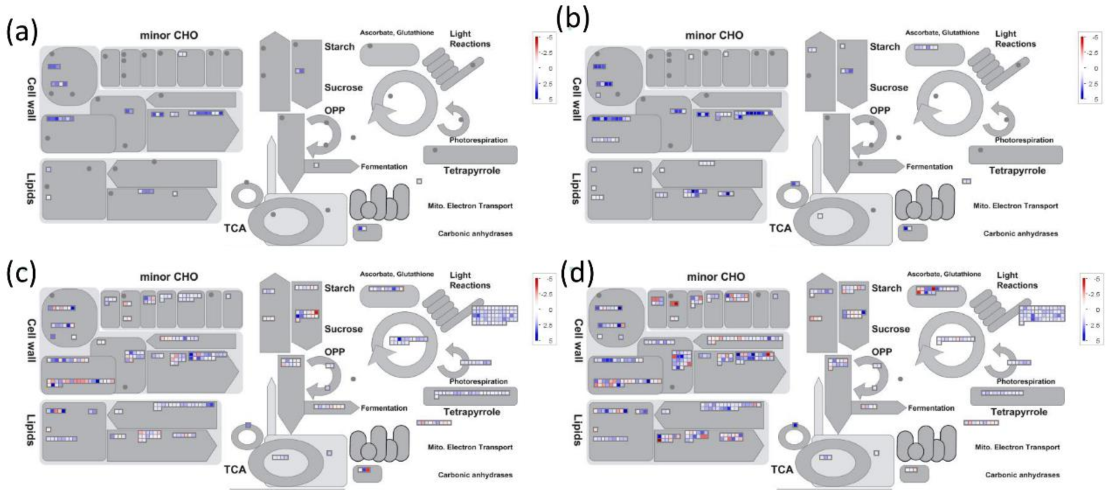

Supplementary Figure S3. The overview of photosynthesis and carbohydrate metabolism in MapMan pathway. (a), the 'photosynthesis and carbohydrate metabolism overview' MapMan pathway was used to visualize the transcriptional changes in pollinated ovaries at 3 DAA. (b), the 'photosynthesis and carbohydrate metabolism' MapMan pathway was used to visualize the transcriptional changes in GA_{4+7} treated ovaries at 3 DAA. (c), the 'photosynthesis and carbohydrate metabolism' MapMan pathway was used to visualize the transcriptional changes in pollinated ovaries at 9 DAA. (d), the 'photosynthesis and carbohydrate metabolism' MapMan pathway was used to visualize the transcriptional changes in GA_{4+7} treated ovaries at 9 DAA.

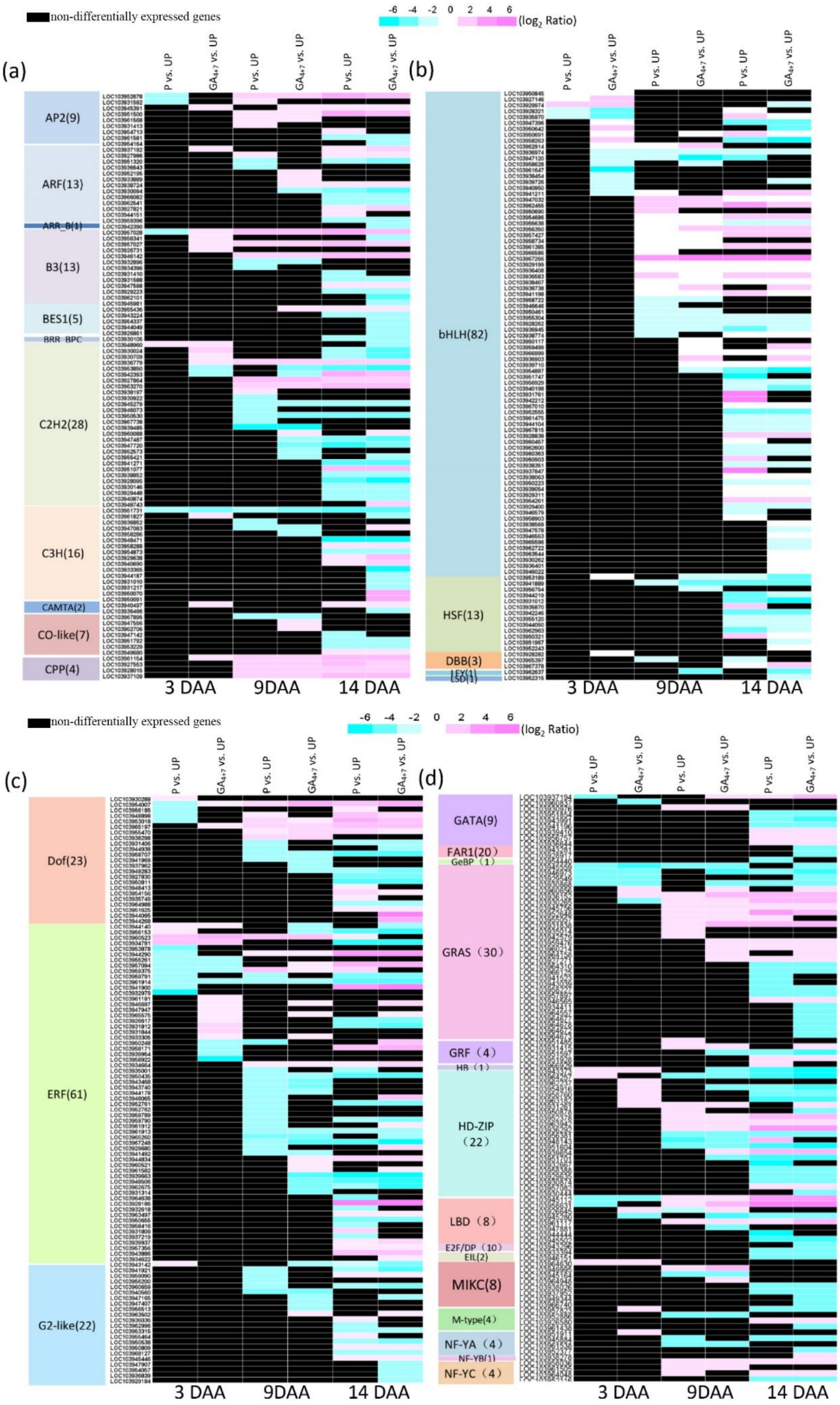
non-differentially expressed genes
-6 -4 -2 0 2 4 6
(log2 Ratio)
(a)
P vs. UP
GA4+7 vs. UP
AP2(9)
ARF(13)
ARR_B(1)
B3(13)
BES1(5)
BBR_BPC
C2H2(28)
C3H(16)
CAMTA(2)
CO-like(7)
CPP(4)
3 DAA
9DAA
14 DAA
(b)
bHLH(82)
HSF(13)
DBB(3)
LFY(1)
LSD(1)
(c)
Dof(23)
ERF(61)
G2-like(22)
(d)
GATA(9)
FAR1(20)
GeBP (1)
GRAS (30)
GRF (4)
HB (1)
HD-ZIP (22)
LBD (8)
E2F/DP (10)
EIL(2)
MIKC(8)
M-type(4)
NF-YA (4)
NF-YB(1)
NF-YC (4)

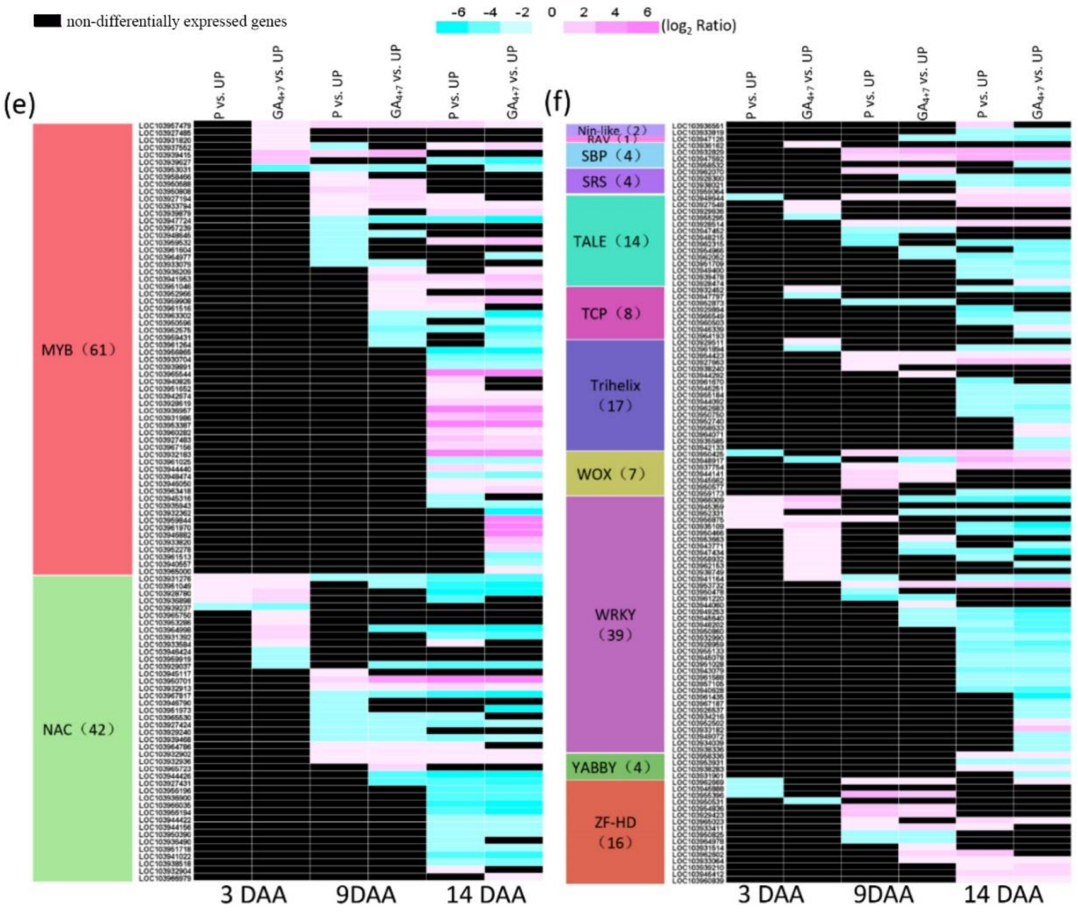

Supplementary Figure S4. The up/down-regulated transcription factors involved in pollination and GA_{4+7} treated ovaries at 3, 9 and 14 DAA. UP, Unpollination; P, Pollination; GA_{4+7}, GA_{4+7} 75 mg L^{-1}.
